# Drug Screening of Human GBM Spheroids in Brain Cancer Chip

**DOI:** 10.1038/s41598-018-33641-2

**Published:** 2018-10-18

**Authors:** Metin Akay, John Hite, Naze Gul Avci, Yantao Fan, Yasemin Akay, Guangrong Lu, Jay-Jiguang Zhu

**Affiliations:** 1 Department of Biomedical Engineering, University of Houston, 3517 Cullen Blvd, Houston, TX USA; 2 Mischer Neuroscience Associates and the Vivian L. Smith Department of Neurosurgery University of Texas Health Science Center in Houston, UTHealth and Memorial Hermann, 6400 Fannin St. Suite 2800, Houston, TX 77030 USA

## Abstract

Glioblastoma multiforme (GBM), an extremely invasive and high-grade (grade IV) glioma, is the most common and aggressive form of brain cancer. It has a poor prognosis, with a median overall survival of only 11 months in the general GBM population and 14.6 to 21 months in clinical trial participants with standard GBM therapies, including maximum safe craniotomy, adjuvant radiation, and chemotherapies. Therefore, new approaches for developing effective treatments, such as a tool for assessing tumor cell drug response before drug treatments are administered, are urgently needed to improve patient survival. To address this issue, we developed an improved brain cancer chip with a diffusion prevention mechanism that blocks drugs crossing from one channel to another. In the current study, we demonstrate that the chip has the ability to culture 3D spheroids from patient tumor specimen-derived GBM cells obtained from three GBM patients. Two clinical drugs used to treat GBM, temozolomide (TMZ) and bevacizumab (Avastin, BEV), were applied and a range of relative concentrations was generated by the microfluidic channels in the brain cancer chip. The results showed that TMZ works more effectively when used in combination with BEV compared to TMZ alone. We believe that this low-cost brain cancer chip could be further developed to generate optimal combination of chemotherapy drugs tailored to individual GBM patients.

## Introduction

The National Institute of Health predicted that 23,800 cases of brain cancer were expected to occur in the United States in 2017^1^. Of all types of brain cancer, glioblastoma multiforme (GBM), a highly invasive cancer, is the most common, accounting for approximately 54% of brain cancer^1,2^. GBM recurs at a high rate after tumor resection and adjuvant therapies, including radiation and chemotherapy with temozolomide (TMZ), with a median overall survival of only 11 months in all GBM patients^3–5^. With the current standard of care (maximum safe craniotomy, adjuvant radiation and chemotherapy with TMZ for newly diagnosed GBM and bevacizumab (BEV) for recurrent GBM) for all GBM patients, variable patient responses to drug treatment are common, often due to inter- and intra-tumor heterogeneity and subsequent genetic mutations which confer drug resistance^6,7^. Therefore, an urgent need exists for a new method of determining the most effective drug treatment regimen for each patient (e.g., mono or multi-chemotherapies, optimal drug concentrations, etc.). To this end, our aim is to develop low-cost technologies that can simulate an *in vivo* environment, allowing us to culture primary cells from GBM patients in 3D and test a range of concentrations for each drug.

GBM tumor growth is characterized by a proliferative outer region, a hypoxic core, and abnormally permeable and leaky vasculature^8,9^. Conventional methods for testing the effectiveness of cancer treatments *in vivo* are based on data collected using 2D adherent cell culture. However, this environment is unlike the tumor microenvironment inside the body, as it fails to recapitulate the complex cell-matrix and cell-cell interactions *in vivo*^10–13^. Research on cancer drug response has historically been performed using commercially available cell lines and 2D cell culture. However, *in vivo* cellular responses to tumor treatments are often different from *in vitro*, and cell lines are poor substitutes for primary patient-derived cells^14^. The effectiveness of 3D cell culture techniques has been tested and verified extensively in recent years, and is considered a viable alternative to traditional cell culture methods^15–18^. The 3D culture techniques have also recently been combined with high-throughput methods for drug screening and assessment^19,20^.

As described in a recent study^21^, we fabricated a brain cancer chip based on microfluidics technology and demonstrated its ability to culture spheroids from a commercially available GBM cell line (U87). It was shown that upon entering the microwells, the cultured cells formed 3D spheroids within 7 days. Furthermore, the concentration gradient created by the multiple microfluidic channels allowed for delivery of two commercially available cancer drugs to the U87 spheroids at varying concentrations. We tested the effectiveness of the platform by using pitavastatin and irinotecan in the brain cancer chip. We were encouraged by our preliminary data indicating that the combination of the two drugs is more effective in reducing the size of U87 spheroids, compared with either in isolation.

While the results of our previous study based on the U87 cell line were very promising—showing the effectiveness of our brain cancer chip at culturing cancer cells in 3D spheroids and its ability to apply drugs in combination—the investigation of human samples in the current study required us to modify the platform to make it more clinically relevant. Indeed, one challenge to overcome was the diffusion of drug molecules through the porous hydrogel matrix. Small molecules are able to permeate through the hydrogel, creating the potential for contamination of drug concentration across channels. Furthermore, while cell lines such as U87 are very useful in many applications, including early-stage drug testing and culture array analysis, they are more resilient in experimental settings compared to primary human or animal-derived cells, in part due to the cumulative molecular mutations^21^. Additionally, the behavior of U87 cells *in vivo* has been shown to differ from primary cancer cells in mouse xenograft model studies^22,23^. Therefore, it was necessary to culture cells extracted from tumors obtained from patients diagnosed with GBM in the brain cancer chip, so as to assess the ability of the chip to provide patient-specific drug response data.

In this study, we aim to show the ability of the brain cancer chip to culture primary, tumor-derived human GBM cells as stable, viable cancer spheroids. The chip was also improved to prevent any small drug molecules from diffusing across channels, thus removing the potential for cross-channel interference. Small ‘diffusion gaps’ in the hydrogel now prevent small molecules from passing from one channel to another. Two new drugs that are commonly prescribed to treat GBM were selected for use in the brain cancer chip: temozolomide (TMZ, trade name Temodar) and bevacizumab (BEV, Avastin). TMZ is an alkylating agent that methylates DNA at the O6 or N7 positions of guanine residues, interfering with DNA replication and then triggering cell death^24–27^. TMZ, along with radiation therapy, is one of the most commonly prescribed treatment options for GBM, and is regarded as the gold standard for GBM treatment. However, despite its status as the primary treatment for GBM, a significant portion of the population have normal expression of MGMT gene to MGMT protein, which is a repair enzyme which confers resistance to TMZ^24,25^, highlighting the need for rapid feedback to patient response to TMZ. BEV is a monoclonal antibody for VEGF-A that acts as an angiogenesis inhibitor *in vivo* and is regularly used to treat GBM in combination with other treatments, for recurrent GBM^28,29^. In this paper, we cultured primary, human-derived GBM tumor cells as 3D spheroids in an improved brain cancer chip, and tested the effectiveness of the platform for a possible treatment of human samples using a combination of clinically used drugs.

## Results

### Brain cancer chip design

**Figure 1:**
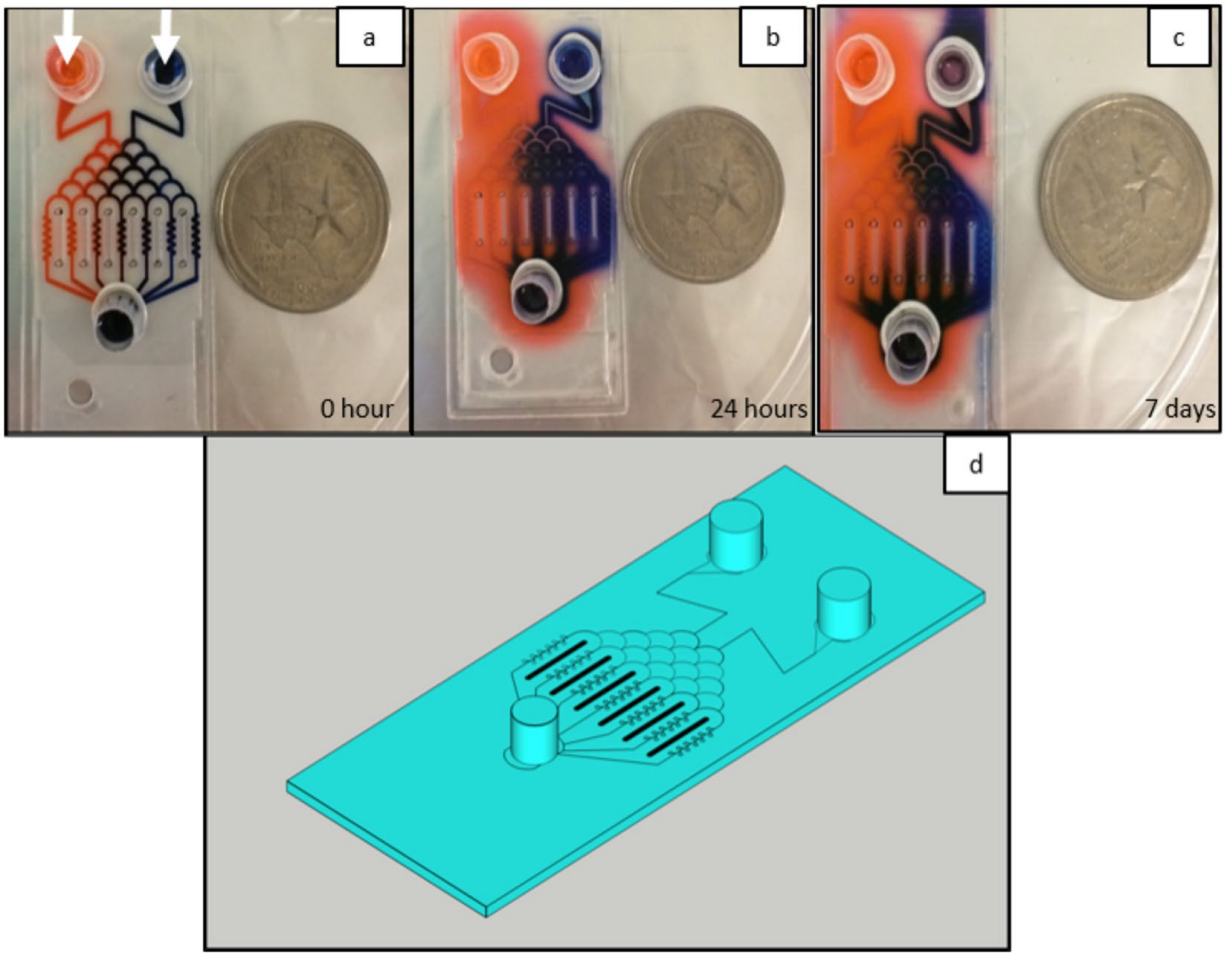
Eosin red (left) and Chicago blue (right) dyes were loaded into the inlets to characterize the gradient of two solutions generated in the microfluidic channels. The dyes were left in the chip for 24 hours to diffuse into the hydrogel, but was unable to cross the gradient generating gap between channels. Gradient generating gaps are marked with white arrows (**a**). The layout of the brain cancer chip flows solution loaded into the two inlets, through a gradient generating regions, and into 7 microfluidic channels, each containing a culture array composed of 9–11 microwells 360 µm in diameter (**b**). The image was taken at day 7 to test the leakage (**c**). The coins are a US Quarter.

Our updated brain cancer chip is composed of a hydrogel layer in between upper and lower cover glass slides that have been treated for increased attachment. The hydrogel solution is composed of 20% (v/v) poly-(ethylene glycol) diacrylate (PEGDA, MW 700 Da) in phosphate-buffered saline (PBS), and is formed using photolithography via controlled exposure to UV light under a patterned photomask. To increase the number of microwells in the chip, our new design consists of two inlets and a single outlet, connected by 7 microfluidic channels with 9–11 microwells per channel. The microwells are 360 µm in diameter, while the microfluidic channels are 100 µm in diameter, narrowing to 50 µm at the opening of each microwell to increase cell capture efficiency. The design of our channels generates a concentration gradient across the chip when two different solutions are loaded to the left and right inlets, respectively, and this concentration gradient is used to measure the combinatorial effect of drugs at varying relative concentrations (Fig. 1a–d).

As previously reported^21^, small molecules such as drug molecules dissolved in solution can easily diffuse through the porous hydrogel matrix. In practice, this diffusion could allow drug molecules to migrate from one channel to another. To avoid this kind of diffusion, there is a 600 µm wide diffusion-prevention gap (Fig. 1d) in the solid hydrogel between each microfluidic channel in the culture array section of the microfluidic chip, for a total of 6 diffusion-prevention regions. These open gaps do not affect the flow of the microfluidic channels and serve only to prevent small molecule diffusion between channels. To test the effectiveness of these gaps, Chicago Blue dye was applied to the right inlet and Eosin red dye to the left and both were allowed to flow through the microfluidic channels (Fig. 1a) and then diffuse through the hydrogel overnight (Fig. 1b). As the images indicate, the dye was unable to cross through the diffusion-prevention regions. Thus, this diffusion gap allows our chip to effectively separate the 3D culture arrays in each channel from those in other channels, as drug solutions from one channel cannot cross over into another. In order to test for any leakage, the microchip was kept for seven days and an additional image was taken (Fig. 1c).

### Human GBM cell growth in brain cancer chip

In order to apply our system to patient-derived primary GBM cells, we collaborated with clinicians in University of Texas Health Science Center at Houston (UTHealth) and Memorial Hermann, Texas Medical Center, Houston, Texas.

**Figure 2:**
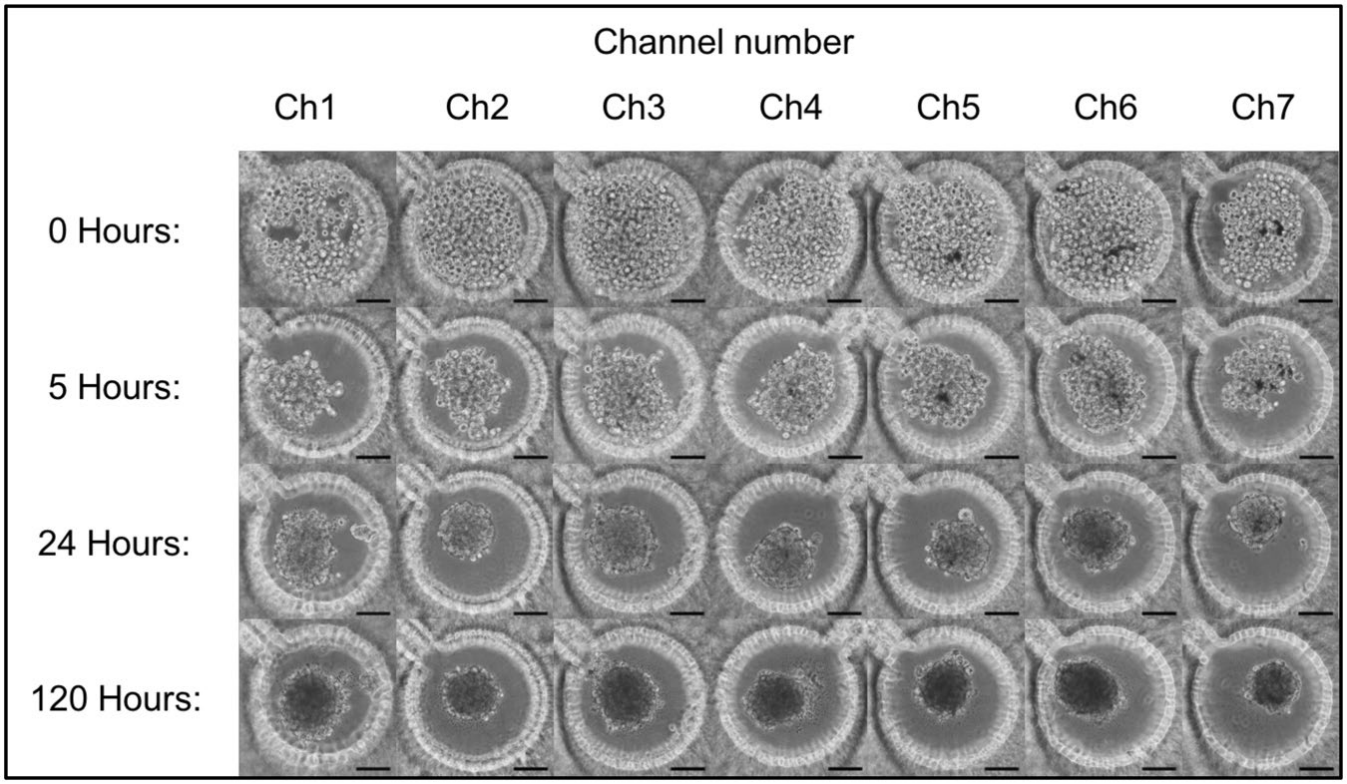
Cells were seeded into the microfluidic chips at 0.5 × 10^6^ cells/mL and captured in the microwells. Images were taken 0, 5, 24, and 120 hours after cell seeding to visualize cell aggregation and spheroid formation. Scale bar is 100 µm.

The project was approved by both human subject research protection committees at UTHealth and University of Houston, and informed consent for participation in this study was obtained from each subject. All methods were performed in accordance with the relevant guidelines. Fresh primary human GBM specimens were acquired from three patients operated at Memorial Hermann and UTHealth. The tumor tissue was dissociated and cells were cultured in Endothelial Growth Medium - 2 (EGM-2). EGM-2 allows for the growth of multiple cell types, including cancer cells, endothelial cells, and others, effectively reproducing the heterogeneous cell make-up of *in vivo* tumors. We first attempted to use freshly resected tumor cells in the brain cancer chip. However, in our preliminary study, the cell viability of the fresh tumor cells in the chip was poor (data not shown). Therefore, to avoid poor cell viability in the brain cancer chip, as suggested by Tsai *et al*.^30^ we cultured the cells outside of the chip using established tissue culture protocols, and then inserted the cells in the chip. These cells were initially cultured in 2D tissue flasks and passaged 1–5 times before being seeded into the brain cancer chips. To seed cells into the chip, cells from each patient were suspended in EGM-2 cell culture media and loaded into both inlets simultaneously. Cells from each of the three patients were seeded onto brain cancer chips three times, for a total of 9 chips. As previously demonstrated^20^, as well as seen in Fig. 2, the gradient-generator region of the brain cancer chip directs the cells equally across the microfluidic channels as cell-containing media flows through the chip towards the outlet, and cells were captured in microwells across the chip.

**Figure 3:**
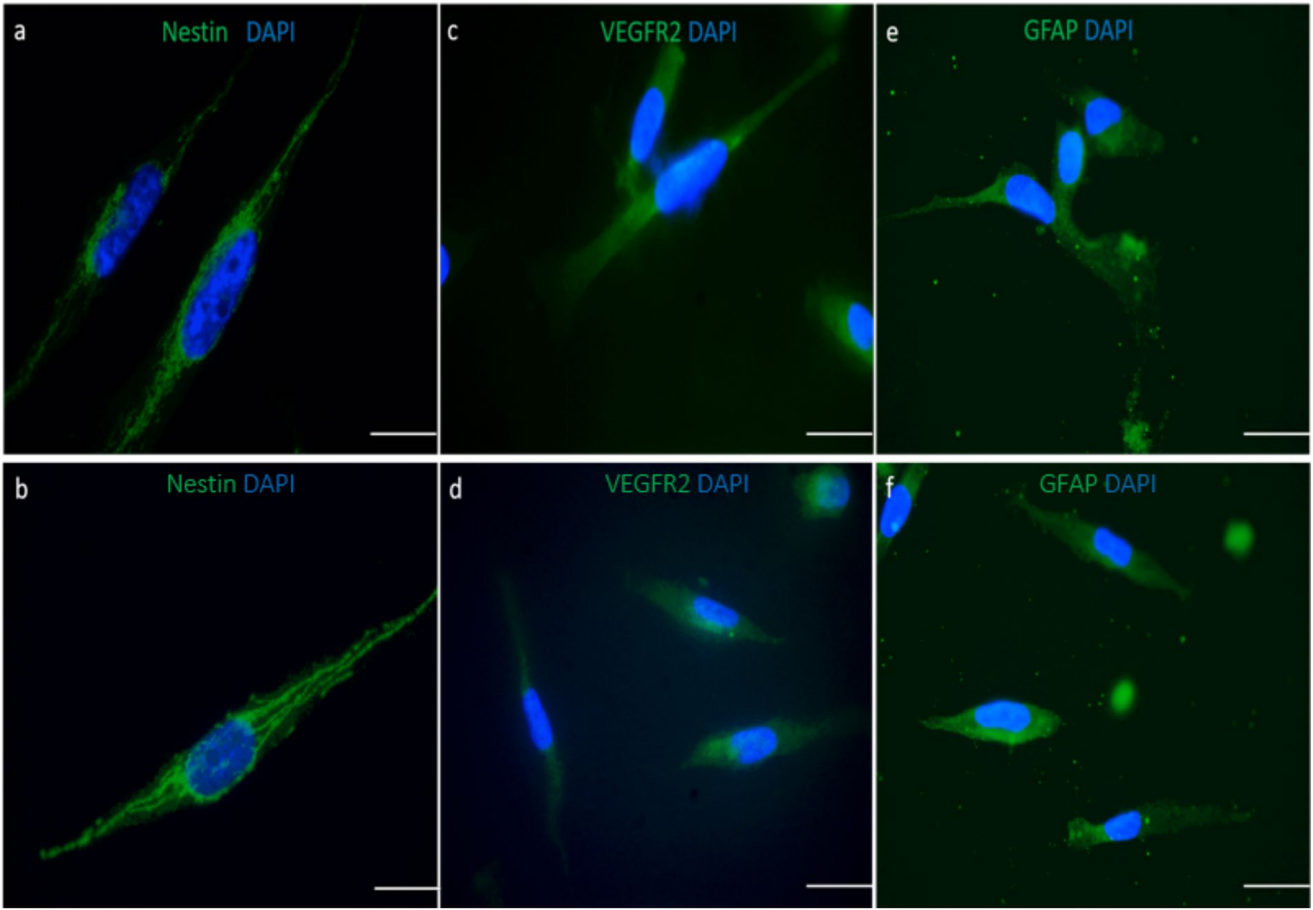
Characterization of the cells collected from freshly dissociated cells (**a,c,e**) and from the microfluidic chips (**b,d,f**). Nestin (**a,b**), VEGFR2 (**c,d**), GFAP (**e,f**) stainings of the cells extracted from fresh primary tumor and cells collected from microfluidic chip, respectively. Cells were seeded on the coverslides, incubated overnight to attach to the coverslides and stained against the antibodies. Nuclei are stained with DAPI. Scale bar is 100 µm.

To characterize the primary tumor cells, their immunoreactivity for GBM markers was detected. For the analysis of nestin, vascular endothelial growth factor receptor-2 (VEGFR2) and Glial Fibrillary Acidic Protein (GFAP) expression, immunofluorescence assay was used. Both freshly dissociated tumor cells and cells collected from microfluidic chips showed higher expression of nestin. Nestin-positive intermediate filaments formation were detected in both cultures in cells with similar morphology. In the freshly dissociated cells, a small number of weak nestin-expressing cells were observed. After passage 4, most of the cells in the culture were nestin positive (Fig. 3a,b). Weak signal of the surface-presented and cytosolic VEGFR2 were observed in both cultures (Fig. 3c,d). The astrocytic feature of the GBM cells was showed by GFAP-positive cells. Although small proportion of the cells exhibited diffuse signal for GFAP, higher expression of GFAP was observed in a majority of both cells (Fig. 3e,f).

After seeding the cells into the brain cancer chips and observing the cell capture within the microwells across all 7 channels, the seeded chips were cultured for 7 days at 37 °C in the brain cancer chip and were monitored to ensure proper spheroid formation. The process of spheroid formation is shown in Fig. 2, with images from each channel taken immediately following the cell seeding, as well as after 5, 24, and 120 hours, respectively. Within 5 hours of seeding, cells began to migrate towards each other in the center of the microwells. Cancer spheroids formed within 1–3 days of 3D culture and continued to increase in cell density over the remaining culture time. Media was replaced with fresh media every 2–3 days in the microwells by removing 100 µl from the outlet and applying 50 µl to each inlet. Slow speed and low volumes were used to prevent excessive sheer stress from a high flow rate from damaging the spheroids during the initial culture. A trypan blue exclusion assay was performed on day 7 and the average cell viability of day 7 spheroids was assessed to be 95 ± 1.87%.

### Drug response assessment

To investigate the ability of the chip to evaluate drug effectiveness on GBM cells, we chose to test two FDA-approved drugs used to treat glioblastoma patients clinically, TMZ and BEV. After 7 days of 3D culture, TMZ and BEV were applied to the cancer spheroids through the right and left channels, respectively. The drug solutions were prepared in EGM-2 media, and 7.5 µM BEV solution was applied to the left inlet, while 600 µM TMZ solution was applied to the right inlet.

**Figure 4:**
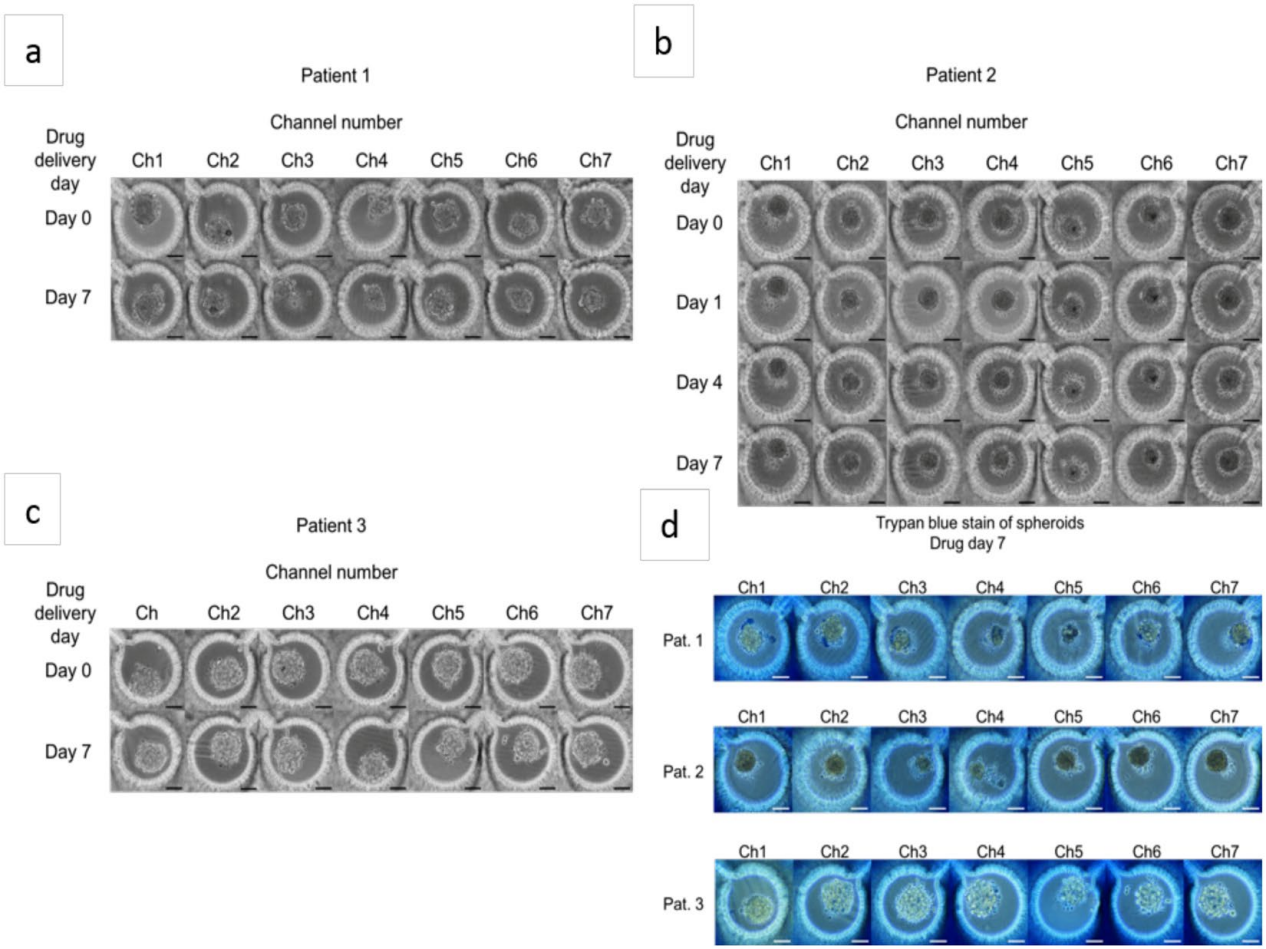
After 7 days of spheroid culture, TMZ (600 µM, right inlet) and BEV (7.5 µM, left inlet) were applied to the brain cancer chip. Effects of the drug treatment on the cancer spheroids were visualized by the shrinking spheroid volume and the disaggregation of dead cells from the spheroids in patient 1 (**a**), patient 2 (**b**), and patient 3 (**c**). Seven days after drug administration, the cells were briefly rinsed with PBS and loaded with 0.4% trypan blue for semi-quantitative cell viability (**d**). Quantitative analysis was performed off chip using a trypan blue exclusion. Scale bar is 100 µm.

**Figure 5:**
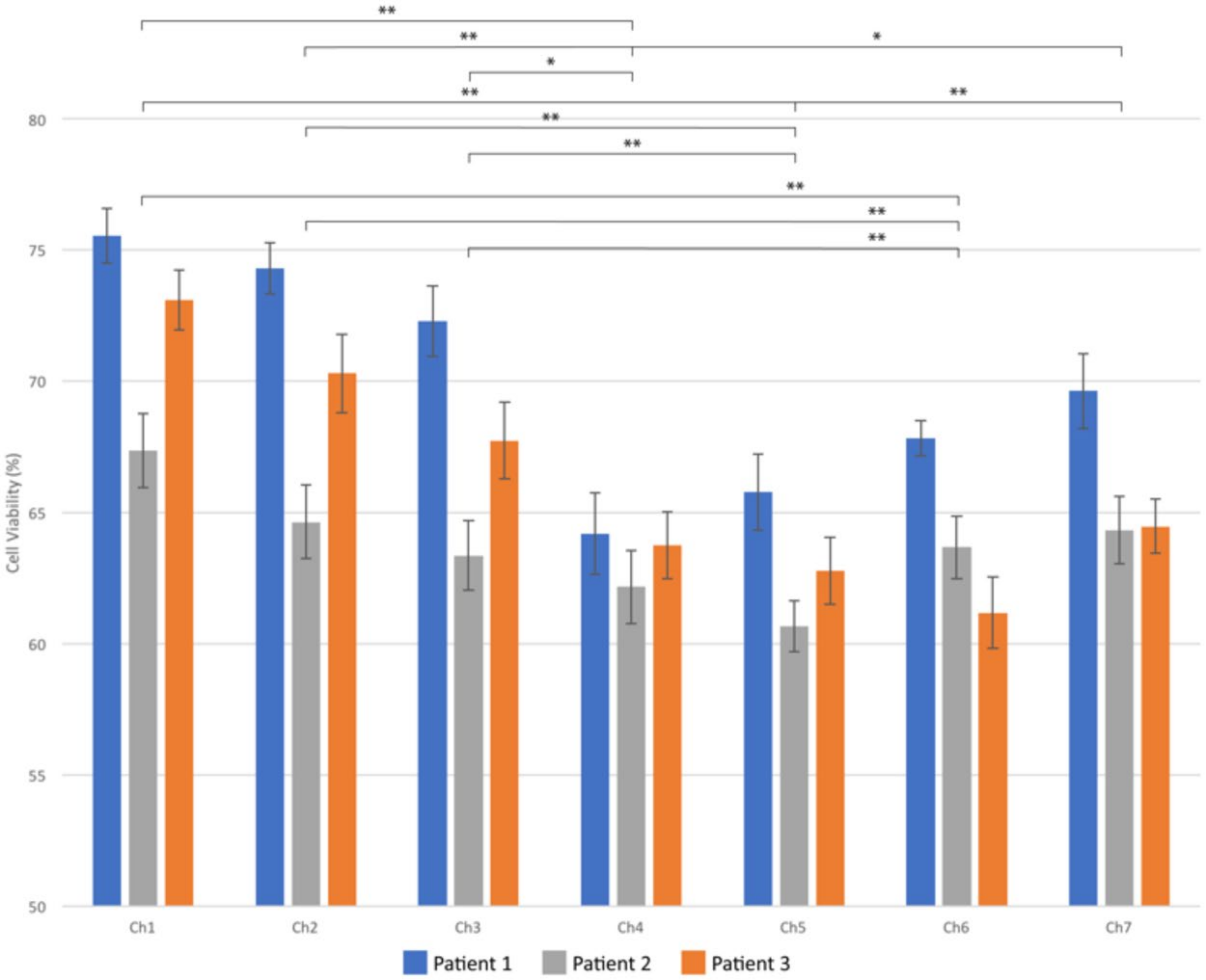
Cell viabilities in each channel are given for patients 1, 2 and 3. A single factor ANOVA test was used to calculate p-values. ^*^Denotes p ≤ 0.05 and ^**^denotes p ≤ 0.01.

The effectiveness of these drugs on the three patients was measured over the course of 7 days (Fig. 4a–c). Semi-quantitative analysis of the images shows the effect of the combinatorial drug regimen. Spheroids began to shrink as cells died and broke away from the spheroids. On day 7 after drug administration (day 14 of overall culture on chip), a trypan blue exclusion assay was performed to assess spheroid viability. Quantitative analysis of cell viabilities revealed a differing response between patients. As can be seen in Fig. 5, Patient 1 showed the highest drug effect on cell viability in channel 4, with 64.20 ± 1.55% cell viability, compared to 75.56 ± 1.05% in channel 1 (p < 0.000001) and 69.63 ± 1.43% in channel 7 (p < 0.02). Patient 2 showed the highest response in channel 5, with 60.66 ± 1.31% viability, compared to 67.35 ± 1.14% in channel 1 (p < 0.001) and 64.33 ± 1.24% in channel 7 (p < 0.03). Patient 3 had the highest drug response in channel 6, with 62.18 ± 1.19 in channel 6, and 73.11 ± 1.40 in channel 1 (p < 0.0000001) and 64.47 ± 1.26 in channel 7. Control (non-drug treated) cell viabilities for day 14 for patient 1 were 90.52 ± 1.01%; patient 2: 89.84 ± 0.89%; patient 3: 91.92 ± 0.53%. There is a clear difference in drug response between each patient, confirming variability in drug response between individuals. However, in all three cases, a combinatorial drug regimen resulted in a higher instance of cell death than either single drug treatment, although in the case of patient 3 the difference was not statistically significant when compared to TMZ treatment alone.

When analyzing the combined results from all three patients, we observed the most effective combinations of drugs to occur in channel 4 and channel 5, which demonstrate 63.74 ± 0.81% and 63.34 ± 0.75% cell viabilities, respectively. The average cell viability of channel 1 spheroids over all patients is 72.15 ± 0.77% (Ch1-Ch4, p < 1 × 10^−11^; Ch1-Ch5, p < 1 * 10^−13^), while in channel 7 it is 66.37 ± 0.46% (Ch4-Ch7, p < 0.02; Ch5-Ch7, p < 0.005). Additionally, these results demonstrate that TMZ alone (channel 7) is more effective at treating human GBM cells than BEV alone (channel 1) (p < 0.000001).

## Discussion

In this paper, we both expanded upon our previous microfluidic brain cancer chip and demonstrated its effectiveness in culturing human GBM cells as cancer spheroids. We implemented diffusion prevention regions that impede the transmission of small drug molecules across channels. These diffusion gaps, seen in Fig. 1, ensure that the drug combinations in each channel are governed only by the microfluidic gradient generation of the chip. We then cultured primary cancer cells isolated from tumors extracted from three patients diagnosed with GBM as 3D cancer spheroids and assessed their response to varying concentrations of two different clinical cancer drugs, TMZ and BEV.

The brain cancer chip was able to maintain the genetic fidelity of the primary tumors. The immunofluorescence staining of the GBM tumors with tumor specific markers evaluated the impact of the brain microchip’s microenvironment on the expression of Nestin, VEGFR2 and GFAP. Upregulated nestin expression has been detected in different brain tumors and tumors derived from CNS tissues, such as pilocytic astrocytomas, and malignant gliomas including glioblastoma multiforme^31,32^. We characterized the morphology of nestin-positive filaments in the cytoplasm of both cell cultures as a distinct network of intermediate filaments. This finding has been confirmed by the literature as well^31^. Tumor microenvironment has to be taken into consideration for the tumor therapy since it influences GBM treatment outcome. As one of the most angiogenic tumors, neovascularization in GBM tumors is regulated by VEGF and VEGFR2^33^. This receptor is used as a target in the development of antiangiogenic drugs (e.g., bevacizumab, sunitinib, sorafenib, etc)^32^ and its expression may vary with tumor grade^33^. Despite the weak signal, our results showed similar levels of VEGFR2 expression in both cell cultures. The weak expression of the VEGFR2 in GBM primary tumors can be explained by the expression differences in tumor grades. Even in the most aggressive glioma tumors, the expression levels of VEGFR2 may show differences^34^. GFAP, a marker for astrocytes, expression was also similar in both cell cultures regardless of passaging numbers.

The brain microchip was also capable of culturing human-derived cancer cells as 3D cancer spheroids. This is an important step in the development of the brain cancer chip, as it indicates that the chip provides a stable platform to culture not only resilient cell lines, such as U87, but also primary cancer cells extracted from primary human GBM specimens. With this capacity, the chip holds great promise as a tool for clinicians to deliver rapid, personalized information regarding the efficacy of multiple treatment options in a clinical setting.

The result of drug administration revealed differing drug responses between the three patients tested, though with a common trend towards a higher cell killing effect of both drugs in combination as compared to individual drug treatment, and with a stronger effect of TMZ compared to BEV. When assessed for cell viability 7 days after drug treatment, each patient had a slightly different response. The highest levels of cell death for each patient were in the following channels: patient 1, channel 4; patient 2, channel 5; and patient 3, channel 6. This differing response is unsurprising, as patient-to-patient variability in glioblastoma treatment has been well documented^7,24–27,35^. This variability in patient response to treatment is a key factor limiting the ability to treat GBM, and emphasizes the need for a platform to provide patient-specific assessment of drug treatment options. Based on our encouraging preliminary data, we believe that our brain cancer chip could fill this role in the treatment of GBM.

We also analyzed the overall drug response across all three patients to confirm the average behavior of the GBM cancer spheroids. Based on the combined drug response data, the overall most effective combination of drugs occurred in channels 4 and 5, corresponding to approximately 300 µM TMZ and 3.75 µM BEV and 360 µM TMZ and 3 µM BEV, respectively, based on previously reported flow quantification^21^. There was an apparent stronger effect of TMZ alone when compared to BEV alone, which was anticipated based on the mode of action between the two drugs. TMZ is an alkylating agent targeting DNA and triggering cell death^4,24^, while BEV functions as an angiogenesis inhibitor, and primarily serves to aid in the functionality of other drugs, such as TMZ and irinotecan^28,29^.

Our brain chip has great potential as both a tool for oncologists to provide rapid information regarding patient specific drug response and as an instrument for use in early stage testing of new drugs as a replacement for 2D cell culture. In its current state, the platform can be used to provide rapid results in a clinical setting. If paired with a high-throughput image analyzer, the chip could give more in-depth analysis of drug combination responses with very little input from the technician, making this a low-cost, simple method for developing personalized treatment plans. In the future, we plan to further these results by increasing the drug database used in the brain microchips by studying the genetic fidelity of the cultured primary tumor cells and the effects of other clinical drugs, as well as validating the platform for the culture of other types of cancer, such as breast and ovarian cancer.

## Methods

### Fabrication of microfluidic brain cancer chip

In order to fabricate the device, cover glass slides (24 mm × 60 mm, Corning) were treated with 3-(Trimethoxysilyl)propyl methacrylate 98% (TMSPMA) to generate an adhesive surface suitable for crosslinking to the PEGDA hydrogel, as previously reported^20^. The slides were bathed in a 10% NaOH solution overnight, then washed with distilled water and 100% ethanol repeatedly. Once dry, they were soaked in TMSPMA at 70 °C overnight, then washed with 100% ethanol again and baked at 100 °C overnight. A glass coverslip slide was prepared using a 100 W CO_2_ laser cutter (CAMFive). Two inlets, a single outlet, and six pairs of vent holes for uncross-linked hydrogel removal were cut into the top glass slide, and a filling port was cut into the bottom slide. Reservoirs were affixed to the inlets and outlets to provide space for PBS or culture media to be loaded. An acrylic frame 500 µm in thickness was cut to shape using the same CO_2_ laser, and the frame was placed between two treated cover glass slides. The liquid hydrogel solution was prepared by dissolving monomeric PEGDA (20% v/v, Polysciences, Inc., Warrington, PA) and a photoinitiator, 2-Hydroxy-4-(2-hydroxyethoxy)−2-methylpropiophenone (PI, Sigma Aldrich, St. Louis, MO) in D-PBS. One mL of the solution was loaded into the chip frame through the filling port and a photomask was aligned to the inlets, outlet, and the holes cut for the diffusion prevention gaps. The photomasks were designed using AutoCAD (AutoDesk, Inc.) and printed onto a plastic surface with a high level of transparency (CADart, Bandon, OR). Photo-polymerization occurred via UV exposure with an Omnicure S2000 (320–500 nm, EXFO, Ontario, Canada) lamp at 100 mW/cm^2^ at a distance of 16 cm for 15.0 seconds. Once the hydrogel was cross-linked, uncross-linked hydrogel was removed from the diffusion-prevention gap via vacuum pump, and the microfluidic channels were washed with sterile PBS. After the uncross-linked PEGDA was removed, the chip was placed upright and exposed to UV light for 15.0 seconds again to crosslink any remaining unlinked hydrogel solution. The fabricated chips were stored at 37 °C until they were loaded with human sample cells.

### Leak testing and flow characterization

To confirm the flow characteristics of the microfluidic chip, Chicago Blue dye (Sigma) and Eosin Y dye (Sigma) were dissolved in PBS to a concentration of 5 µM to generate blue and red dye solutions, respectively. The red dye was loaded into the left inlet and the blue dye was loaded into the right inlet. To show the effectiveness of the diffusion prevention regions, blue dye was loaded into both channels and allowed to diffuse for 24 hours. Images were captured at 0 and 24 hours.

### Human GBM cell extraction and culture

We obtained three resected glioblastoma tumors from the UTHealth and Memorial Hermann, Texas Medical Center. The project was approved by both human subject research protection committees at UTHealth and University of Houston, and informed consent for participation in this study was obtained from each subject. All methods were performed in accordance with the relevant guidelines. The tumors were dissociated and extracted cells were cultured in 2D tissue culture flasks. The tumors were placed in Endothelial Basal Medium supplemented with hEGF, hydrocortisone, GA-1000, fetal bovine serum (FBS), VEGF, hFGF-B, R^3^-IGF-1, and ascorbic acid (EGM-2, Lonza, Basel, Switzerland) on ice for transport from the surgical facility to our laboratory at the University of Houston. In a sterile laminar flow hood, the tumors were rinsed with PBS, then cut into small pieces with autoclaved scissors before being digested with Accumax (STEMCELL Technologies Vancouver, Canada) for 30 minutes. The digested tissue was pipetted up and down before being passed through a 100 µm cell strainer, followed by a 40 µm cell strainer. The cell solution was spun down in a centrifuge at 300 g for 3 minutes and the cell pellet resuspended in EGM-2, then loaded into T25 cell culture flasks treated for increased attachment (VWR International, Radnor, PA). The human cells were stored in a cell culture incubator at 5% CO_2_, 37 °C, and 95% humidity. Cell culture media was changed every 2–3 days and cells were passaged when they reached 80% confluence.

### Human GBM cell seeding and spheroid formation in chip

Human GBM cells were cultured in 2D tissue culture flasks for 3–5 passages, the cells were seeded into the brain cancer chips. Based on previously gathered data and initial preliminary testing, cells were seeded into the brain cancer chips at 0.5 × 10^6^ cells/mL. EGM-2 cell culture media containing GBM cells was loaded into both inlets simultaneously, 100 µL at a time. Cells were captured in the microwells in each channel and cultured for 7 days. Cell culture medium was changed every 2–3 days by removing 200 µL of media from the outlet and adding 100 µL of media to both inlets. This was repeated 3 times to flush the old media through and replace it with fresh media. Images of the cells were taken immediately, 5, 24, and 120 hours after cell seeding.

### Immunofluorescence assay

Briefly, slides were fixed with 4% PFA for 10 min, blocked with 1% BSA/10% normal serum in 0.1% PBS-Tween20 for 30 minutes and immunostained with mouse anti-nestin (Abcam), rabbit anti-VEGFR2 (Abcam) and rabbit anti-GFAP (Abcam) overnight at 4 °C. Alexa 488-conjugated goat anti-mouse and anti-rabbit antibodies (Molecular Probes, Invitrogen, France) were added as secondary reagents. Nuclei were counterstained with DAPI. Samples were subjected to evaluation under a fluorescence microscope.

### Drug Administration

After 7 days of spheroid culture in the brain cancer chip, a combination of drugs was applied to the chip. Bevacizumab, trade name Avastin (BEV, Roche, Basel, Switzerland) was prepared at a concentration of 7.5 µM in EGM-2 media^36^. Temozolomide (TMZ, Sigma Aldrich, St. Louis, MO) was dissolved in dimethysulfoxide (DMSO) to prepare a solution of 10 mM TMZ^37^. This solution was further diluted to 600 µM TMZ in EGM-2 media. Cell culture media was removed from the chip and replaced by adding 7.5 µM BEV to the left inlet and 600 µM TMZ to the right inlet 100 µL at a time, repeated 4 times^38–40^. Drug administration occurred only once, and cells were left in the brain cancer chip for 7 days following drug administration. Control (non-drug treated) brain cancer chips were maintained under the same conditions.

### Quantification of cell viability

For a rapid, semi-quantitative assessment of cell viabilities, the spheroids were stained with 0.4% trypan blue on the chip. In order to quantify the viability of the spheroid after drug administration, the spheroids were removed from the chip and washed twice with PBS, then digested with trypsin to dissociate them. The cells were stained with 0.4% trypan blue solution, and cell counts were performed with a hemocytometer. Each channel contained 9–11 spheroids and cells from each patient were seeded onto 3 chips.

### Statistical analysis

Statistical analysis of collected data was done using a single-factor analysis of variance (ANOVA), followed by two-tailed Student’s t-test. Confidence intervals were set at 95% (p < 0.05). Error bars are mean ± standard error.
